# Spectrally monitoring the response of the biocrust moss *Syntrichia caninervis* to altered precipitation regimes

**DOI:** 10.1038/srep41793

**Published:** 2017-02-06

**Authors:** Kristina E. Young, Sasha C. Reed

**Affiliations:** 1 U.S. Geological Survey, Southwest Biological Science Center, 2290 S. West Resource Blvd. Moab, UT 84532, USA

## Abstract

Climate change is expected to impact drylands worldwide by increasing temperatures and changing precipitation patterns. These effects have known feedbacks to the functional roles of dryland biological soil crust communities (biocrusts), which are expected to undergo significant climate-induced changes in community structure and function. Nevertheless, our ability to monitor the status and physiology of biocrusts with remote sensing is limited due to the heterogeneous nature of dryland landscapes and the desiccation tolerance of biocrusts, which leaves them frequently photosynthetically inactive and difficult to assess. To address this critical limitation, we subjected a dominant biocrust species *Syntrichia caninervis* to climate-induced stress in the form of small, frequent watering events, and spectrally monitored the dry mosses’ progression towards mortality. We found points of spectral sensitivity responding to experimentally-induced stress in desiccated mosses, indicating that spectral imaging is an effective tool to monitor photosynthetically inactive biocrusts. Comparing the Normalized Difference Vegetation Index (NDVI), the Simple Ratio (SR), and the Normalized Pigment Chlorophyll Index (NPCI), we found NDVI minimally effective at capturing stress in precipitation-stressed dry mosses, while the SR and NPCI were highly effective. Our results suggest the strong potential for utilizing spectroscopy and chlorophyll-derived indices to monitor biocrust ecophysiological status, even when biocrusts are dry, with important implications for improving our understanding of dryland functioning.

Covering almost 45% of the terrestrial surface^1^, drylands – arid, semiarid, and dry subhumid regions where precipitation is infrequent and variable^2^ – comprise Earth’s largest terrestrial biome^3^. Moreover, these expansive ecosystems are expected to undergo significant shifts in community composition and nutrient cycling under future climate^4^,^5^, with global-scale consequences for carbon (C) and nutrient cycling^6^,^7^. The ability to capture climate-induced changes to fundamental dryland communities via remote sensing would represent a dramatic advance in our capacity to consider and predict biotic responses to global change. However, capturing relevant ecological information in arid and semiarid systems has proven a significant challenge, and remote sensing techniques that work in more mesic ecosystems are not always successful in drylands^8^. Specifically, remote sensing indices often lack the sensitivity for high accuracy in sites with low vegetation cover, such as in many arid and semiarid ecosystems, and the indices themselves can be influenced by the highly variable background soils common in drylands^9^,^10^.

Importantly, frequently used biophysical and vegetation indices are often unable to accurately capture ecological and distributional information on the foundational communities of biological soil crust (biocrust) organisms living on myriad dryland soil surfaces. Biocrust communities of mosses, lichens, cyanobacteria, fungi, and algae are fundamental members of drylands worldwide^11^, enhancing soil stability^12^, and in some cases aiding in water retention in these dry systems^13^. Biocrusts also directly influence C and nitrogen (N) cycling^14^,^15^, and thus an improved ability to monitor biocrust extent and response to environmental change would significantly increase our understanding of dryland biogeochemical cycling.

Much of the difficulty in successfully monitoring biocrusts with commonly used biophysical and vegetation indices is centered around biocrust organisms’ limited periods of photosynthetic activity, which occur only when the organisms are wet. While biocrusts can look similar to green vegetation during wet periods, during the majority of time when biocrusts are dry, they appear spectrally similar to bare soil. Indeed, the “desert artifact”, or unexpectedly high Normalized Difference Vegetation Index (NDVI) values in plant-devoid dryland regions^16^ have been attributed to the spectral reflectance of hydrated biocrust organisms^17^. While still widely used within the field of biocrust monitoring, NDVI has had, in some cases, minimal success in monitoring biocrusts in the absence of precipitation^18^,^19^.

Because of the importance of biocrust organisms to dryland ecosystem processes, spectrally differentiating biocrusts from bare soil and vegetation is an active area of research. Biocrust mapping indices were developed by Karnieli *et al*.^20^, Chen *et al*.^16^, Weber *et al*.^21^ and Chamizo *et al*.^22^, to respectively map cyanobacterial crusts, lichen dominated crusts, general biocrust communities, and biocrust type, with a focus on development stage and degree of disturbance. Additionally, the reflectance spectroscopy of biocrusts has been increasingly explored. Spectral characteristics across biocrust types have been observed in the 667–682 nm region of chlorophyll absorption^23^, at 1450 nm and 1920 nm attributed to water or hydroxyl bonds^9^,^24^, and around 2300 nm assigned to the presence of lignin or cellulose^25^. Further, examining the spectral characteristics of specific types of biocrusts have indicated numerous absorption features. Spectroscopy employed to determine ecophysiological parameters of biocrusts has successfully differentiated between crusts in different seasons^25^, explored the spectral response of different watering regimes^18^, and examined the effects of disturbance^22^.

However, despite the important information attained from mapping indices and reflectance spectroscopy, noticeably lacking are points of spectral sensitivity and indices targeting the ecological progressions and functions associated with biocrusts. Specifically, the ability to spectrally monitor biocrust functional response to climatic change is increasingly important^26^, as a growing body of literature suggests these organisms will experience significant shifts in both function and composition, resulting in large scale changes to soil stability, soil fertility, and biogeochemical cycling under future climate^5^,^15^,^26^,^27^,^28^,^29^.

To address this critical gap in our understanding, here we assessed absorbance features and spectral indices for a dominant biocrust moss while subjecting some of the moss samples to experimental, climate-induced changes in physiological and ecological functioning, and leaving other moss samples as controls. Our goals were to: 1. Examine absorption features that can be used to monitor the functioning of biocrust mosses when the mosses are dry; 2. Evaluate and compare spectral indices, such as NDVI, to determine if they capture climate-induced physiological stress within biocrust mosses when mosses were dry; 3. Assess narrowband chlorophyll-specific vegetation indices to test our capacity to monitor chlorophyll degradation in dry, stressed mosses; and 4. Explore the effects of moss stress on N cycling and soil fertility across a timeline of stress. Through these lines of investigation, we aimed to assess the relevance of spectroscopy to monitoring biocrusts’ response to global change.

## Materials and Methods

### Selection of our model biocrust organism

In both natural ecosystems^30^ and controlled field experiments^26^, small (<1.5 mm), frequent precipitation events have caused rapid mortality in the dominant biocrust moss *S. caninervis*, thought to be a consequence of C starvation^27^. The changes in wetting frequencies result in chlorotic moss leaves that have decreased chlorophyll a levels and a higher xanthophyll to chlorophyll a ratio^30^. We focused on *S. caninervis* in part because it is the dominant biocrust moss on the Colorado Plateau^28^ and is widely distributed throughout northern hemisphere drylands^31^. In addition, because C starvation and chlorophyll degradation events represent obvious physiological changes to the functioning of *S. caninervis*, and because these stress and mortality events have been experimentally shown to alter N cycling in the soil matrix beneath the mosses^26^, we chose *S. caninervis* as a model organism to spectrally monitor the progression towards mortality as we applied small (1.25 mm) frequent (twice weekly) watering events. The choice of *S. caninervis* as a model organism is timely, as some climate models predict smaller, more frequent precipitation events will replace the historic monsoon-driven patterns of the American Southwest^32^,^33^, potentially disproportionally affecting this dominant biocrust community member.

### Inducing climate derived physiological stress on S. caninervis

Intact *S. caninervis* samples (n = 61) were collected from a well-developed biocrust community near Moab, Utah on the Colorado Plateau, USA. To aid in collection, mosses were dampened with deionized (DI) water, and upon collection the moss and underlying 1 cm of soil was placed into plastic petri dishes measuring 1.5 cm deep and 8.5 cm in diameter. Once in the petri dish, samples were placed in a greenhouse for the duration of the experiment. The greenhouse was always within ~+5 °C of the outside air temperature, which ranged from 1.2 °C to 46.5 °C over the course of the experiment. Average photosynthetically active radiation (PAR) was 80.23 W/m^2^, measured on a PAR sensor adjacent to the moss samples. After collection, all samples were randomly assigned a treatment and destructive harvest date.

After leaving the moss undisturbed in the greenhouse for one-week following field collection to provide a settling period, we began watering treatments and spectral monitoring, which occurred from July – November, 2013 (lasting 19 weeks). During this time, we maintained two treatments: Control, which received no watering and Watered, which received a watering treatment in line with treatments that induced C starvation and mortality at nearby sites^26^,^27^. Specifically, Watered moss received deionized (DI) water additions simulating a 1.25 mm rainfall event. This amount was one quarter of the average event size based on four years of climate data compiled by Coe *et al*.^29^. Simulated rainfall events were administered using a hand spray bottle every Tuesday and Thursday for the duration of the experiment (19 weeks). The frequency of application mimicked Reed *et al*.^28^, which induced rapid mortality in *S. caninervis* in a climate manipulation field experiment. Control and Watered moss samples were randomly placed on a table in the greenhouse and re-randomized monthly to account for micro-climate variations within the greenhouse. To replicate *in situ* desiccation conditions, after watering occurred, all samples (including controls) were put outside the greenhouse for two hours, and were then placed back in the greenhouse. Outside placement did not occur when it was raining or when the outside temperature was significantly cooler than the temperature inside the greenhouse.

### Spectrally monitoring dry S. caninervis samples

Spectral data were collected using an ASD FieldSpec^®^ 3 Hi- Res portable spectroradiometer. Once a week for the duration of the experiment, spectral assessments were made of all *S. caninervis* samples using a Leaf Contact Probe, which maintained contact with the sample during spectral readings. Full spectra (350 nm–2500 nm) were collected on dry mosses while the samples were in the greenhouse immediately before the watering treatment was applied to ensure the moss was in its most desiccated state. Before each set of measurements, a white reference was taken and was repeated every 20–30 minutes. Five readings for each sample were taken without moving the probe, then averaged together to correct for variations and steps (abrupt changes of the recorded reflectance) that occur due to sensitivity drift of the instrument. Because of the homogenous appearance of the samples, the small size of the petri dishes, and the large number of replicates at the start of the experiment, we believe the experimental design accounted for spatial variability within the samples. Spectral data files were exported as ASCII text using the ASD ViewSpec Pro software, and stored with the meta-data in an excel file.

### Choosing Spectral Indices

We chose to examine the effectiveness of the NDVI to monitor stress in *S. caninervis*, as NDVI is one of the most widely used broad band spectral indices, and has been utilized extensively for vascular plant and biocrust assessment in drylands^18^,^34^,^35^,^36^. Additionally, we examined narrow- band indices aimed at detecting chlorophyll content to determine if these indices effectively captured chlorophyll loss in dry *S. caninervis* samples. We chose to examine the Simple Ratio Index (SR) as described by Gitelson & Merzlyak^37^, and the Normalized Pigment Chlorophyll Ratio Index (NPCI) as described by Penuelas *et al*.^38^ (Table 1), both of which are commonly used and sensitive to leaf chlorophyll content.

### Biogeochemical measurements

The experiment began with 61 moss samples: 25 Control samples, 25 Watered samples, 5 samples used for pre-treatment analysis, and 6 reserved for post-experiment analysis. We treated those 61 samples as follows: to elucidate how N cycling beneath *S. caninervis* changed over time in response to small frequent watering events, we harvested five Watered and five Control samples every 3.5 weeks, for a total of six harvests over the course of the experiment, with five additional samples harvested immediately after initial sample field collection to capture pre- treatment patterns. Three Control and three Watered Moss were left un-harvested to retain for visual assessment at the end of the experiment. For all soil biogeochemical analyses, we removed moss samples from the petri dishes and homogenized the moss and underlying soil sample with a mortar and pestle. The samples were then sieved through a 2 mm sieve, which allowed soil particles to pass through, but captured the vegetative body of the moss. Extractable soil ammonium, nitrate, and total inorganic N concentrations were assessed in the following way: ~10 g (dry weight equivalent) sieved soil were extracted in 35 ml of 2 M KCl, shaken for 1 hour, and left to sit for 18 hours. The next day the extract was filtered using a vacuum filter manifold and 0.45 μm Millipore filters^37^. Liquid soil extracts were assessed for N using a SmartChem autoanalyzer (Westco, Inc.) to determine concentrations of nitrate (NO_3_^−^) and ammonium (NH_4_^+^). Total inorganic N was calculated as the sum of NO_3_^−^and NH_4_^+^.

### Statistical Methods

All data were tested for assumptions of normality and homogeneity of variance (using Levene’s test for the equality of variance) and, when assumptions were violated, data were ln transformed prior to statistical analysis. Data transformation consistently resolved problems and transformed data were normally distributed and had homogenous variance. We used a full- factorial repeated measures general linear model (GLM) to explore the effect of sampling date and watering treatment on spectral indices (18 measurement time points). We selected a repeated measures approach because the same samples were being measured through time but we note that, due to the need for destructive harvests, there were a different number of samples measured over the course of the experiment. We propose that the use of repeated measures is appropriate for three key reasons. First, the same number of samples was measured for each treatment at each time step (i.e., destructive harvests didn’t preferentially remove samples from either treatment). Second, residual plots show no change in data variance through time (data not shown). Finally, if we didn’t use repeated measures we couldn’t account for temporal autocorrelation and sample self-similarity through time, and would thus violate assumptions of sample independence. We also used repeated measures GLM to compare moss spectral changes through time in Control and Watered moss that were not destructively harvested, focusing on 450 nm, 540 nm, 665 nm, 680 nm, 695 nm, and 720 nm wavelengths. In contrast, we did not use repeated measures assessments for the analysis of the biogeochemical data because they were not the exact same samples being measured through time. For the biogeochemical statistical analysis we used a multivariate GLM, with time and watering treatment as fixed factors.

When there was a significant interaction between date and treatment, treatment effects were also examined within each sampling date using t-tests (Table 2). Although repeated measures assessment of NDVI data did not suggest a significant interaction between date of sampling and treatment, inspection of the data showed patterns of treatment variability among dates and t-tests were thus also performed on NDVI data for each date individually. For all analyses, significance was determined at ✓ = 0.05 and all statistical analyses were conducted in SPSS (v. 21; IBM, Armonk, NY).

## Results

**Figure 1:**
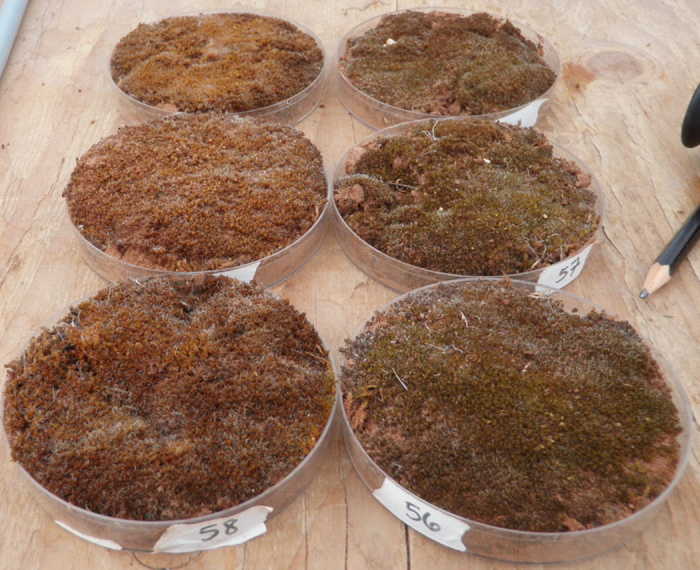
Pictures of moss samples: Watered (left) and Control (right) *S. caninervis*, wetted with 1.25 mm of water after 19 weeks of treatment. This photo captures the chlorotic state of the reddish Watered mosses at the end of the experiment compared to the green, photosynthetically active state of the Control mosses. We note that while moss shown here were wetted to highlight their visible difference, spectral measurements were made on moss samples when they were dry.

The Watered treatment samples of *S. caninervis* showed visible signs of reddening or chlorosis - a symptom of chlorophyll degradation - when the samples were wet, starting in week 10 of the experiment and increasing over time. No signs of chlorosis were visible when the Watered samples were dry. The Control *S. caninervis* samples, which never received water, remained in their desiccated state for the entirety of the experiment and never showed visible signs of chlorosis. A visual comparison of the level of reddening between the two treatments was made at the end of the experiment. This was done by giving three Control mosses and three Watered mosses 1.25 mm of water after the experiment was completed. Chlorosis was visible in the Watered mosses, while the Control mosses immediately turned green with no signs of chlorosis (Fig. 1). This visible difference in moss tissue color is an indication that our experimental watering application was successful in inducing physiological stress and chlorophyll degradation in *S. caninervis* and that, as has been shown many times (e.g. ref. 39), while being left dry did not have negative effects on the moss.

**Figure 2:**
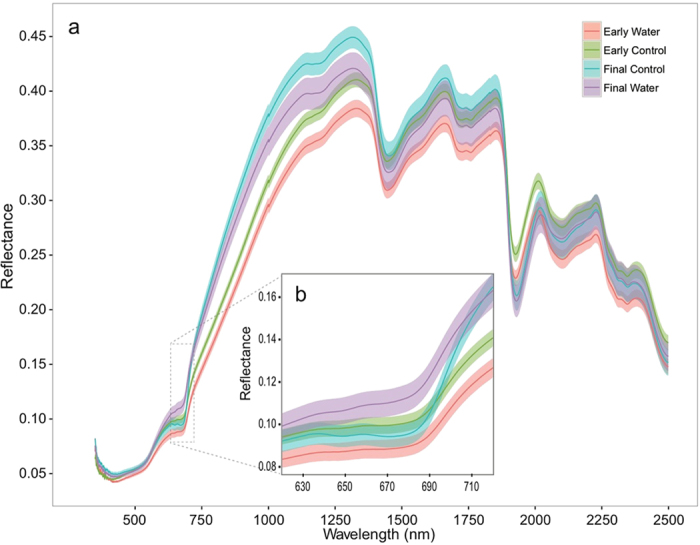
(**a**) Full reflectance graph of dry *S. caninervis* samples near the beginning and end of the 19-week experiment. Data shown are for Control samples assessed at the end of week 1 of the experiment (Early Control; n = 50), Watered samples at the end of week 1 of the experiment (Early Water; n = 50), Control samples at the end of the entire treatment period (week 19) (Final Control; n = 5), and Water samples at the end of the entire treatment period (week 19) (Final Water; n = 5). Reduced sample size at the end of the treatment period is due to destructive harvests made throughout the experiment in order to assess biogeochemical soil properties. Solid lines represent mean values, and colored ribbons represent standard error surrounding the mean. **(b)** The same graph zoomed in to the 620 nm–720 nm range, highlighting differences between the final Water and all of the other treatments in the 680 nm region of chlorophyll detection, and the abrupt rise of the final Control samples at the red edge (>700 nm).

Within the visible spectra, effects of watering on mosses were most apparent in the 650 nm –720 nm region (Fig. 2), with Watered samples demonstrating higher reflectance values. Comparing Watered samples at the beginning of the experiment (Early) with Watered samples at the end of the experiment (Final) show differences in the 650 nm–720 nm region not seen when comparing the early Control and final Control samples. When examining specific wavelengths associated with pigmentation, 665 nm, 680 nm (absorption maximum of chlorophyll a), 695 nm, and 720 nm exhibited significantly higher reflectance values in the final readings of Watered samples compared to early readings of the same moss samples (Table 2). In contrast, there were no significant differences among the sampling times for Control mosses at these wavelengths. There was also no change through time for any samples, regardless of treatment, at the 450 nm and 540 nm (VIS green) wavelengths. Both the final Watered and final Control samples in the near-infrared exhibit higher reflectance values than the early Control and early Watered, and while it is only statistically significant for the Watered samples (Table 2), the Control samples showed P values near significance (p = 0.052).

### Effectiveness of spectral indices in detecting *S. caninervis* physiological stress and chlorophyll degradation

**Figure 3:**
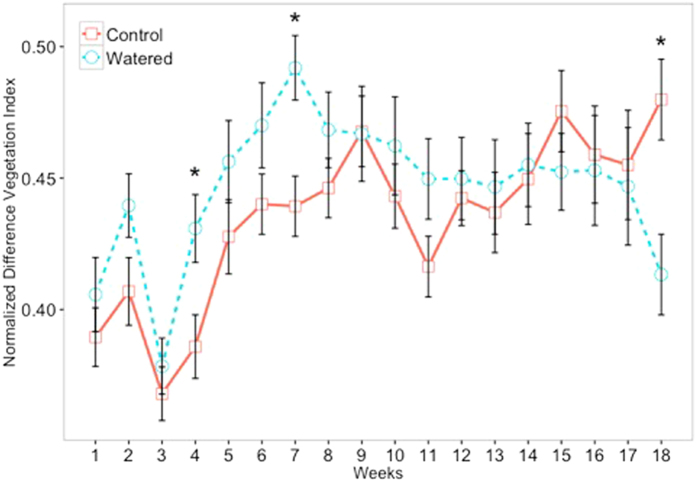
Normalized Difference Vegetation Index (NDVI) values over time in Control and Watered moss. Data were taken weekly over the course of the 19-week experiment and spectral images were always collected while mosses were dry. Asterisks (*) indicate dates where there were significant differences among treatments as assessed by t-test.

Overall, NDVI values did not statistically vary between treatments, (p = 0.833) (Table 3). NDVI values did vary among sampling time points (p = 0.001), but there was no significant interaction between treatment and time (p = 0.213) (Table 3). Some individual time points showed significant differences between Control and Watered mosses, indicating NDVI is detecting some differences between treatments (Fig. 3), however, this detection does not mirror the strong patterns of reddening and chlorophyll degradation visible in the Watered moss treatment (Fig. 1).

**Figure 4:**
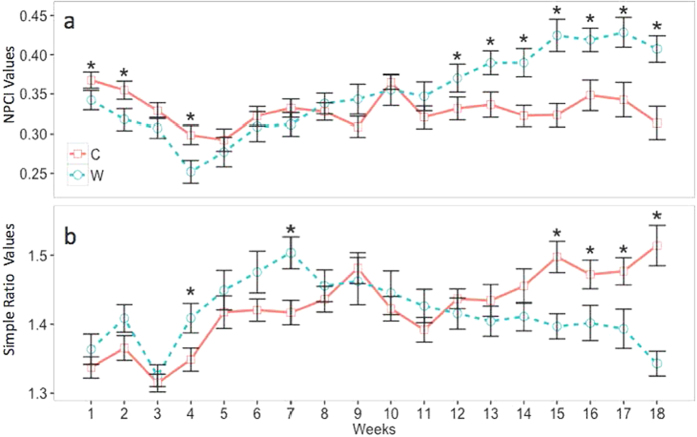
Index values calculated from spectral assessment of dry mosses for the Control and Watered *S. caninervis* samples. Asterisks (*) indicate dates when there were significant differences among treatments as assessed by t-test. **(a)** Normalized Pigment Chlorophyll Ratio Index (NPCI) showed that Watered moss samples had more total pigments/chlorophyll relative to the Control samples. NPCI is inversely related to chlorophyll content, so samples with a higher chlorophyll content will have a lower NPCI index value^54^. **(b)** Simple Ratio (SR) showed increased reflectance in points sensitive to chlorophyll a content, and therefore decreasing values for the ratio suggest lowered chlorophyll a content.

The narrow-band SR and NPCI both showed significant change over time and between treatments (Table 3). For the Watered samples, the SR index rose significantly at week 4 (p = 0.031), and then significantly declined at week 15 (p = 0.002) and remained reduced through the final 3 weeks of the experiment. This pattern was consistent with patterns in NPCI, where differences between treatments were significant at week 4 (p = 0.016) and then again from week 13 (p = 0.023) through week 18 (Fig. 4).

### Biogeochemical Assessments

**Figure 5:**
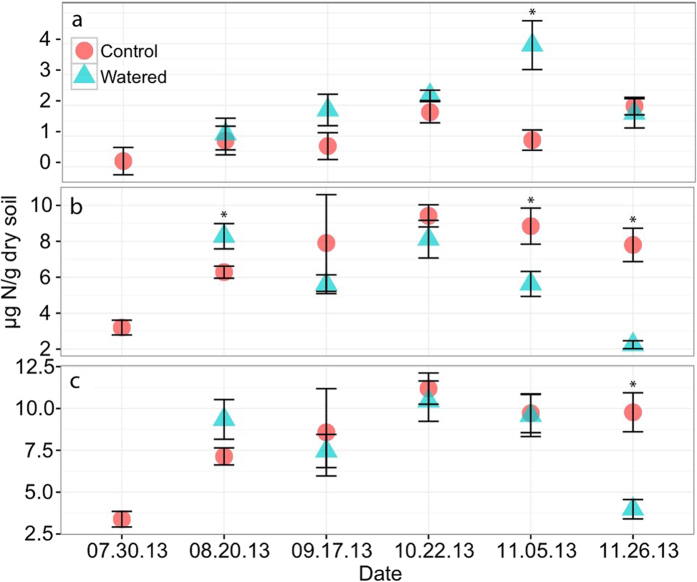
Changes in soil N cycling over time beneath climate-stressed mosses. Changing levels of: **(a)** soil extractable nitrate concentrations (ug NO_3_^−^/g dry soil), **(b)** soil extractable ammonium concentrations (ug NH_4_^+^/g dry soil), and **(c)** estimated available soil N concentrations (NO_3_^−^ + NH_4_^+^) in Watered and Control moss over the 19-week duration of the experiment. Asterisks (*) indicate significant differences as assessed by t-tests.

Watering treatments resulted in reduced levels of soil extractable NH_4_^+^ in the soil beneath *S. caninervis* samples (Fig. 5). In particular, while soil extractable NH_4_^+^ concentrations were initially ~7 μg NH_4_^+^/g dry soil, at week 12 (10.22.13) NH_4_^+^ began steadily declining to ~2 μg NH_4_^+^/g dry soil in the Watered samples. In contrast, except for the pre-treatment assessment, Control sample soil NH_4_^+^ concentrations remained between 6 mg and 10 μg NH_4_^+^/g dry soil for the entirety of the experiment. Nitrate levels also varied across the course of the experiment, however, treatment differences were only significant at week 15 (11.05.13) (p = 0.007) and were not significantly different at the final time point (p = 0.681). Throughout time, inorganic N (NH_4_^+^ + NO_3_^−^) concentrations remained relatively consistent between treatments, until the last time point (week 18, 11.26.13) when soil inorganic N declined sharply in the Watered samples (3.98 μg Inorg N/g dry soil) but not in the Control samples (9.77 μg Inorg N/g dry soil) (p = 0.002).

## Discussion

Examination of the spectra provided informative insight into differences in reflectance of unstressed vs. stressed mosses. Increased reflectance in the 650 nm to 700 nm region of dry, physiologically stressed *S. caninervis* compared to that of unstressed moss indicated chlorophyll a degradation, as 680 nm represents a reflectance minimum or absorbance maximum, for chlorophyll a. This absorption feature has been observed across biocrust types^23^. The decreasing chlorophyll levels in the Watered mosses as chlorophyll is lost over time is likely due to disruptions in the thylakoid membranes and, ultimately, a breakdown of chlorophyll a during the repeated movements into and out of desiccation during watering events^39^,^40^.

The lack of differences in reflectance values between early and late Control and Watered mosses in the 400–500 nm region implies we did not capture changes in the absorption of other pigments, such beta-carotene, carotenoids, or phycoerythrin, which have been observed in cyanobacterial dominated crusts^17^,^21^,^22^,^41^. Because we would have expected to see changes in these pigments in concert with declines in chlorophyll^30^, we recognize the utility of coupling the analysis of multiple pigments with spectral assessment to determine the effects of climate change or other perturbation on leaf pigment content and evaluation by remote sensing techniques. Additionally, while it has been shown that the wavelengths relate to these pigments in cyanobacteria dominated biocrusts, there is need for confirmation that the 400–500 nm wavelength region represents the absorbance feature for the above pigments in moss-dominated biocrusts. Based on these findings, it appears the 680 nm region is well suited for detecting climate-induced stress within this biocrust moss, as it related directly to function and corresponded to the mechanism of stress: chlorophyll degradation. This region is likely the best region with which to develop a specific index for monitoring function of dry biocrust mosses, and other desiccated biocrust species. In the near-infrared region, the decreased reflectance in the final Watered when compared to the final Control may be due to increasing brown pigments in the Water mosses over time, as suggested by similar decreases in the reflectance of damaged leaves within higher plants around the far red region^42^. However, the lower reflectance values of the early Water and early Control than both the final Water and final Control do not support this hypothesis. We did observe an absorption feature around 1720 nm, which has been found to indicate cellulose and lignin content in moss biocrust^43^, however, there are no clear patterns related to our treatments.

We also observed decreased reflectance around 1920 nm in our control and stressed mosses when compared to our initial time point. This wavelength has been found to serve as an absorption feature for water or –OH bonds in biocrust^24^,^43^. Additional work is needed to determine biocrust moss response to physiological stress in the infrared region, as a broad band response to climate- induced stress could result in a more easily utilized broad band index.

Each of our indices (NDVI, NPCI, and SR) showed significant moss responses to our watering treatments, although the timing and strength of each index’s capacity to capture the response varied dramatically (Figs 3 and 4). It is not wholly surprising that NDVI was not as well suited at capturing climate-induced physiological stress in dry *S. caninervis* samples. As a broad band “greenness” index, the strength of NDVI lies in its ability to quantify the near- infrared region, a region scattered by mesophyll leaf structures, and the red region, a region absorbed by chlorophyll. This quantification allows for the determination of vegetation presence, and, when examined over time, vegetative productivity^34^. However, because detection of chlorophyll absorption is so tightly coupled with photosynthetic activity, the dry, photosynthetically inactive state of *S. caninervis* during spectral readings likely reduced the effectiveness of the index. As *S. caninervis*, and all other dryland biocrust species, spend significant periods of time in a photosynthetically inactive state between precipitation events, these results suggest indices in addition to or instead of NDVI should be used to capture relevant and timely ecological and functional information on biocrusts.

Based on the above identification of sensitivity to stress in the 680 nm region, vegetative indices aimed at determining chlorophyll content appear better suited for quantifying and monitoring climate-induced physiological stress in *S. caninervis*. The lack of significance between the SR and NPCI when just examining the treatments across the entire 19-week examination highlights the healthy state of both moss treatments at the beginning of the experiment, as the treatment effects took significant time to emerge (Figs 3 and 4). For this reason, when time*treatment and individual timepoints are examined, we see the significant divergence between the two indices, beginning around week 11 of the experiment. Indeed, significant differences between the Control and Watered mosses as assessed by both the SR and NPCI indices beginning around week 11 coincide with the visible reddening of Watered *S. caninervis* samples, first noted in week 10. The change through time observed within this experiment highlights the efficacy of using spectroscopy to measure changes to moss physiology over time, as opposed to single point monitoring. For example, continual spectroscopy measurements compared over time could prove effective in documenting biocrust response to precipitation stress in a field setting.

As the NPCI quantifies the ratio of normalized total pigments to chlorophyll a content, increases in this index in Watered mosses imply treatment-induced changes to the chlorophyll a of the leaves similar to those found previously when analyzing the tissue of chlorotic moss^30^. Similarly, the decreasing index value for the SR also implies decreasing chlorophyll content over time, as this ratio has been shown to be directly proportional (correlation r^2^ > 0.95) to chlorophyll a concentration in higher plants^44^.

The above indices may also provide insight into physiological changes in the time leading up to chlorosis in the Watered moss. Both indices suggest a pattern of *increased* chlorophyll a within the Watered mosses around week 4 and 7, suggesting an increase in chlorophyll a content, possibly due to watering initially increasing allocation to photosynthetic machinery. This is followed by a decrease in chlorophyll a around week 11, presumably when C stores begin to run out^27^, and chlorophyll degrades due to the frequent movement into and out of desiccation states with each small watering event. Gaining this insight demonstrates the potential power of these narrow band indices to provide meaningful information about ecological progressions regardless of the direction of change.

The presumed declines in chlorophyll content may also give insight into the N content of the moss tissue. Chlorophyll content is indirectly related to N content^45^ and remote sensing of chlorophyll tends to scale with N, with correlation coefficients (r^2^) ranging from 0.7 to 0.9^46^. Chlorophyll is ~6% N by mass^47^, and ~75% of the total N content of the plant is contained in chloroplast as the enzyme Rubisco and in chlorophyll binding proteins^48^,^49^. Thus, decreases in chlorophyll content imply decreases in N. A proposed mechanism for the previously observed increases in soil nitrification following moss mortality is increased N inputs from moss to soil, resulting in an increase in soil nitrifiers and nitrification rates^26^. Our spectrally-observed decreases in chlorophyll content, the strong relationship between the abundance of chlorophyll and N, and the changes in N cycling we observed lend support to the idea of increased moss N inputs into the system as chlorophyll degrades and N is leached from moss tissue.

Within our extractions, we observed changes to N cycling in the soil matrix. In particular, the decreasing NH_4_^+^ concentrations at week 3 and week 15 in the Watered mosses, and the elevated NO_3_^−^ concentrations at week 15 suggests a progression towards relative NO_3_^−^ dominance in the soil, though these results are not consistent through time, as NO_3_^−^ ultimately decreased back to Control levels. Nevertheless, the results are relevant in the context of field observations showing increasing NO_3_^−^:NH_4_^+^ ratios following watering-induced moss mortality^26^. Importantly, we observed these changes to the N cycle *before* mortality occurred, implying potential changes in N inputs and N cycling much earlier than has previously been reported. These data suggest that the stress being observed via the indices is likely affecting the soil N cycle long before moss death ensues, and could affect biogeochemical cycling without mortality. This also provides a temporally-resolved view of an altered N economy beneath *S. caninervis*, and indicates that moss stress may be setting the stage for the altered N dynamics shown to persist long after moss mortality occurs^26^.

With the persistence of these patterns, a shift in dominance from NH_4_^+^ to NO_3_^−^ would have implications for ecosystem-wide soil fertility. In many soils, NO_3_^−^ is more easily lost in leached and gaseous forms relative to NH_4_^+^, therefore, over longer timelines, higher levels of NO_3_^−^ could result in less total N^50^,^51^. Such shifts in N availability, as well as in the form of N (i.e., NO_3_^−^ vs. NH_4_^+^ 52), would have consequences at multiple scales, as N is believed to be second only to water as the most limiting resource to biological activity in arid and semiarid ecosystems^53^. In this way, climate-induced physiological stress to the dominant biocrust moss could beget further change to ecosystem function.

Because of the observed shifts in *S. caninervis* physiological function and the resultant shifts in N availability, it becomes apparent that effectively quantifying ecological processes is important in this time of rapid environmental change. A clear direction forward would include the spectral monitoring of other biocrust organisms and determination of their spectral response to environmental change. Additionally, the development of indices aimed at amplifying the small but relevant differences in reflectance of stressed biocrusts could enhance the ability to capture and understand important perturbation-driven ecophysiological changes. Finally, further studies on the feedbacks between climate-induced changes to biocrust physiological and ecological functioning and ecological processes and progressions would allow for the spectroscopy-derived information on biocrust function to be used predictively to determine future biocrust survivorship and function.

## Additional Information

**How to cite this article**: Young, K. E. and Reed, S. C. Spectrally monitoring the response of the biocrust moss *Syntrichia caninervis* to altered precipitation regimes. *Sci. Rep.* **7**, 41793; doi: 10.1038/srep41793 (2017).

**Publisher's note:** Springer Nature remains neutral with regard to jurisdictional claims in published maps and institutional affiliations.

## Figures and Tables

**Table 1:** Vegetation indices explored in this study to assess *S. caninervis* stress.

Index	Index Name	Source	Index Formulas (nm)	Details
NDVI	Normalized Difference Vegetation Index	Ichii *et al*. 2001	((Avg(725 to1100)) − (Avg(580 to 680)))/((Avg(725 to 1100)) + (Avg(580 to 680)))	Index of green vegetation cover
NPCI	Normalized Pigment Chlorophyll Ratio Index	Penuelas *et al*. 1993	(R680 − 430)/(R680 + 430)	Total pigments/chlorophyll
SR	Simple Ratio	Gitelson & Merzlyak, 1996	750/700	Insensitive to chlorophyll detection/sensitive to chlorophyll detection

Index name, reference source, formula used to calculate the index, and a brief explanation of each index are provided. Numbers in the index formula represent wavelengths used to calculate the index.

**Table 2:** Mean percent reflectance values standard error values for early Control, early Water, final Control, and final Water at specific wavelengths along the spectra.

Wavelength	Early Control (*n* = 8)	Stderr	Early Water (*n* = 8)	Stderr	Final Control (*n* = 8)	Stderr	Final Water (*n* = 8)	Stderr
450 nm	4.83	0.21	4.24	0.25	5.06	0.23	4.84	0.23
540 nm	6.22	0.39	5.42	0.39	6.14	0.29	6.05	0.32
665 nm	10.32	1.00	8.87*	**0.87**	9.47	0.52	10.96*	**0.64**
680 nm	10.48	1.04	9.02*	**0.89**	9.55	0.52	11.29*	**0.65**
695 nm	11.74	1.02	10.15**	**0.88**	11.66	0.55	13.15**	**0.67**
720 nm	14.62	0.96	12.69***	**0.86**	16.48	0.66	16.32***	**0.82**

Data are from samples that were not destructively harvested over the course of the experiment. Significant differences among sampling dates as assessed by repeated measures general linear model analysis are shown in bold text and with asterisks depicting significance at *p < 0.05; **p < 0.01; ***p < 0.001.

**Table 3:** Statistical results for spectral indices from repeated measures general linear model assessment of the effects of time (different measurement times over the course of the experiment), watering treatment (Control vs. Watered) and the interaction between time and treatment.

	NDVI			SR			NPCI			NO_3_^−^			NH_4_^+^			Inorganic N		
	df	F	P	df	F	P	df	F	P	df	F	P	df	F	P	df	F	P
Time	**17**	**7.6**	**0.001**	**17**	**5.9**	**0.001**	**17**	**6.4**	**0.001**	5	2.2	0.073	**5**	**12.4**	**0.001**	**5**	**3.7**	**0.008**
Treatment	1	0.046	0.833	1	1.5	0.247	1	2.2	0.159	**1**	**9.7**	**0.003**	**1**	**19.0**	**0.001**	1	2.2	0.148
Time* Treatment	17	1.3	0.213	**17**	**2.2**	**0.004**	**17**	**2.8**	**0.001**	**4**	**4.3**	**0.006**	**1**	**8.7**	**0.001**	**1**	**2.8**	**0.036**

Results from the extractable soil N concentrations were assessed using a multivariate general linear model. Time and treatment effects were assessed for the spectral indices (NDVI, SR, NPCI) and for the extractable soil N concentrations (NO_3_^−^, NH_4_^+^, and total inorganic N), as well as interactions between the factors. Degrees of freedom (df), F values, and P values are provided. Significant differences (p < 0.05) are shown in bold text.
